# Resentment Is Like Drinking Poison? The Heterogeneous Health Effects of Affective Polarization

**DOI:** 10.1177/00221465221075311

**Published:** 2022-02-11

**Authors:** Micah H. Nelson

**Affiliations:** 1 University of North Carolina at Chapel Hill, Chapel Hill, NC, USA

**Keywords:** affective polarization, partisanship, political engagement, self-rated health, stress

## Abstract

Affective polarization—the tendency for individuals to exhibit animosity toward those on the opposite side of the partisan divide—has increased in the United States in recent years. This article presents evidence that this trend may have consequences for Americans’ health. Structural equation model analyses of nationally representative survey data from Pew Research Center’s American Trends Panel (n = 4,685) showed heterogeneous relationships between affectively polarized attitudes and self-rated health. On one hand, such attitudes were directly negatively associated with health such that the polarized political environment was proposed to operate as a sociopolitical stressor. Simultaneously, affective polarization was positively associated with political participation, which in turn was positively associated with health, although the direct negative effect was substantially larger than the indirect positive one. These results suggest that today’s increasingly hostile and pervasive form of partisanship may undermine Americans’ health even as it induces greater political engagement.

Researchers have corroborated what most perhaps already perceive about the contemporary sociopolitical environment in the United States: It is charged, partisan, and divisive. Notably, there is mounting evidence that partisan politics have changed over the course of the last three decades such that party affiliation is not merely, or necessarily, a matter of policy preferences but is marked by hostility toward and distrust of members of the opposition. Time-series surveys of eligible voters show that negative sentiment among Democrats and Republicans toward members of the out-party has increased gradually since 1992 (Iyengar et al. 2019). Furthermore, party affiliates today impute negative personal characteristics to members of the out-party (Iyengar, Sood, and Lelkes 2012) and appear to be disinterested in social, romantic, and economic relationships across party lines (Gift and Gift 2015; Huber and Malhotra 2017; Lee 2020). Such “affective polarization” suggests both a turn toward a more personal and confrontational tone in contemporary politics and a heightened salience of partisan identity in other spheres of life.

Although researchers have been keenly interested in understanding the full range of nonpolitical consequences of this trend, there has been a dearth of empirical investigation about the impacts of affective polarization on health. Although scholars have documented its role in coloring partisans’ views toward public health policies and health behaviors (e.g., Allcott et al. 2020; Lerman, Sadin, and Trachtman 2017), few have assessed direct links between affective polarization and health despite a strong theoretical foundation for hypothesizing such relationships.^1^ On one hand, affective polarization may negatively influence health by perpetuating a more stress-inducing, confrontational social and political milieu; indeed, scholars have already proposed that a hostile sociopolitical environment creates unique stressors that are associated with poor health outcomes (Krieger et al. 2018; Williams and Medlock 2017). On the other hand, it may have a positive relationship with health through political participation. This is because negative sentiment toward the opposing party can be a powerful motivator of engagement (Groenendyk and Banks 2014; Iyengar and Krupenkin 2018), which itself may have health-protective effects, possibly by facilitating social ties and a sense of self-efficacy (Ballard and Syme 2016).

This article investigates these potential links between affective polarization and health by analyzing data from a nationally representative survey of Americans. Using structural equation modeling, the present study decomposes the relationships between affectively polarized attitudes, political participation, and health while controlling for measurement error, which is likely to occur when attempting to capture these more abstract constructs. The analysis presented here demonstrates that there are heterogeneous relationships between affective polarization and health. Although affectively polarized attitudes are directly negatively associated with health, they are positively associated with political engagement, which in turn is associated with improved health. However, the positive indirect effect does not outweigh the stronger, negative direct relationship with health. These findings indicate the possibility that even as the polarized political milieu catalyzes greater participation, animosity in politics today may be subverting the public’s health and well-being.

## Background

### Affective Polarization: Conceptualization and Dimensions

Although there is an active debate regarding the degree to which affective polarization is related to and driven by disagreements over policy, the term aims to capture a decidedly nonideological component of modern partisanship. In fact, researchers typically advance a relatively broad conceptualization of affective polarization as marked by a dislike of or animus toward members of the opposing party relative to copartisans (e.g., Hobolt, Leeper, and Tilley 2020; Iyengar et al. 2019; Webster and Abramowitz 2017). In other words, political parties today are not solely polarized according to ideological stances or issue preferences of constituents but, rather, by sentiment.^2^ It is in this spirit that some have portrayed affective polarization as marking a transformation of partisan affiliation into a social identity in which the bias displayed by partisans resembles that along other social boundaries (Iyengar and Westwood 2015; Mason 2018b).

The simplicity of the predominant definition of affective polarization as marked by a dislike of or bias against the out-party arguably belies the existence of multiple distinct dimensions of the construct. These can be derived from the various measures that have been commonly utilized to capture it (Druckman and Levendusky 2019). First, and most consistent with a broad definition of affective polarization, a number of approaches tap into individuals’ global negative evaluation of the out-party and its defects as a whole. This could be conceived as generalized attitudes toward those who affiliate with or lead that group. The most common such instrument is the feeling thermometer, whereby respondents are asked to rate how “warm” or “cold” they feel toward Democrats and Republicans on a scale of 0 to 100 (e.g., Lelkes and Westwood 2017; Orr and Huber 2020). This measure has been included in nationally representative surveys since at least the 1980s and reveals increasing affective polarization in the United States over time. Indeed, between 1992 and 2016, mean out-party feeling has declined from approximately 50 out of 100 (or “neutral”) to roughly 25 (Iyengar et al. 2019). Other similar measures ask respondents to assign specific characteristics to each party as a whole, like hypocrisy, closed-mindedness, intelligence, and patriotism (e.g., Garrett et al. 2014). The balance of negative and positive trait assignments reveals the degree of underlying bias universalized toward the out-party.

Second, scholars frequently discuss a more relational dimension of affective polarization, focusing on the social distance between members of different parties. In theory, if party affiliation truly is a salient social boundary, one might expect it to bleed into the interpersonal and strain social relationships or impede the formation of new social ties among those with diverging party allegiances (Iyengar et al. 2012). Survey instruments that aim to assess this dimension of partisanship ask respondents how they would react in hypothetical situations involving members of the out-party, such as their willingness to befriend, marry, live near, or socialize with Democrats and Republicans (Mason 2018a). As with feeling thermometer ratings, these measures reveal increasing polarization over time. For instance, whereas in 1960 approximately 5% of partisans opposed their child marrying a member of the out-party, in 2010, 33% and 49% of Democrats and Republicans, respectively, reported some level of unhappiness at such a prospect (Iyengar et al. 2012).^3^ Researchers have also utilized behavioral data to assess social contact across party lines, whether within families (Chen and Rohla 2018; Lee 2020), between peers (Chopik and Motyl 2016), or among those searching for romantic relationships (Huber and Malhotra 2017).

Third, the construct is often described as having an emotive dimension. Aside from expressing a “cold” attitude toward the out-party or being unwilling to interact with its affiliates, a partisan could experience strong negative emotions in reaction to the opposing party. Webster and Abramowitz (2017) offer fear and anger as relevant manifestations, using measures of these emotional reactions to Barack Obama’s presidency as markers of affectively polarized attitudes among Republicans. Similarly, Mason (2016) conducts a survey experiment to assess affective responses to partisan messaging by combining participants’ reports of feeling angry, hostile, and disgusted when exposed to fabricated political blog posts. Others utilize self-reports of anxiety in reaction to the opposing party’s standard-bearer as a marker of negative emotive partisanship (McLaughlin et al. 2020). These emotional responses to the out-party are important because they can be linked to the broader hostile political climate in the United States. This is because they may serve to reinforce interparty animosity given that negative emotions, particularly anger, may drive partisan conflict by motivating political incivility (Miller and Conover 2015). More importantly, these negative emotions are key to hypothesizing a link between affective polarization and health and are the focus of the present study for reasons discussed in the following section.

### Theoretical Links to Health

#### Hypothesizing a negative relationship: affective polarization as a sociopolitical stressor

This article proposes stress as the key mechanism through which affective polarization may undermine health. Scholars have long viewed stress exposure as a threat to both mental and physical health, a position that has been consistently corroborated in empirical research (Slavich 2016). Although stressors can manifest in a variety of ways, there is evidence that chronic strains—long-term, relatively low-level recurrent stressors—are particularly damaging (Thoits 2010). This is because they can elicit repeated activation of a biological stress response and, in doing so, cause a gradual “wear and tear” on the body that undermines the efficiency of its systems, resulting in a greater incidence of health problems (Turner 2013:173). For example, such stress exposure has been linked to earlier onset and increased risk of cardiovascular disease, metabolic disorders, and accelerated cellular aging (Danese and McEwen 2012). Furthermore, stress may lead to injurious health behaviors, such as smoking and alcohol use, as coping strategies (Park and Iacocca 2014). Insofar as social contexts shape the distribution of stress across a population (Pearlin 1989), stress is a crucial pathway through which social conditions take a physiological toll.

A number of researchers have proposed that the political milieu within which one lives can cause stress and, consequently, exert a noticeable impact on health. Much of the literature on the health effects of such sociopolitical stressors focuses on the 2016 U.S. presidential election, which some have found to be particularly distressing for Americans belonging to minority groups. This is because the political rhetoric espoused by then-candidate Donald Trump relied heavily on drawing symbolic boundaries that inflamed racial and religious tensions and othered immigrant, Muslim, black, and Hispanic Americans and other marginalized groups (Lamont, Park, and Ayala-Hurtado 2017). In this vein, a number of time-series studies have found that preterm births among U.S. Hispanic women increased in the period following the 2016 election, which has been attributed to greater stress in response to the anti-immigrant and anti-Hispanic discourse featured in the campaign (Gemmill et al. 2019; Krieger et al. 2018). Similarly, using panel data, Garrison, Doane, and Elliott (2018) document large declines across a range of self-reported health and well-being measures among gay men and lesbians in the aftermath of the election. The authors propose that a contributing factor to these results was stress due to the perception among respondents that President Trump’s election created a hostile environment for LGBTQ Americans.

Such health effects may occur more generally among the broader population due to the stress of electoral contests. Mefford et al. (2020), for example, document an increase in acute cardiovascular disease hospitalizations in California immediately after the 2016 election. Similarly, Rosman et al. (2021) show that North Carolina patients with cardiac devices were more likely to experience arrhythmic events during the election. Consistent with the literature examining minority group health, the focus of research documenting these general health effects for the 2016 election suggests a shared sentiment among scholars that many Americans perceived it to be of particularly high stakes and therefore a more stressful, health-threatening experience.

Following this logic, affective polarization may adversely influence health as a distinct sociopolitical stressor. This hypothesized association rests primarily on the negative emotive dimension of affective polarization—the tendency for the out-party to inspire fear, anger, anxiety, frustration, and other such emotions among partisans—that could be linked to stress through a number of pathways. First, there may be a direct relationship between such strong emotions and stress given that negative affect has been found to elicit a biological stress response and may be associated with elevated risk of poor health, particularly when recurrent (Park et al. 2020; Suls and Bunde 2005). Thus, affective polarization could act as a stressor simply by increasing the frequency at which individuals experience anger, frustration, fear, and anxiety. Second, negative partisan emotions could charge politics in such a way that they cause greater stress in daily life. For example, if the out-party is a source of anger or fear, the wielding of power by its elites could raise the perceived stakes of politics and be distressing. Even the prospect alone that the out-party will take control over the levers of power (as in the case of elections) could be an “anticipatory stressor” (DeAngelis 2020; Grace 2020) in which fear of the mere possibility that one’s preferred candidate could lose may induce a stress response. This could be especially likely to occur if the out-party candidate is viewed as a supporter of policies that threaten one’s interests or health or would take the country in an undesired direction. Like other macro-level social stressors, the causes of such potentially deleterious emotions are embedded in large political and social institutions and therefore are not within the average citizen’s direct sphere of control. Thus, affective polarization may manifest as a chronic strain that repeatedly activates one’s stress response as political events pass into an individual’s awareness and stoke negative emotional states.

It should be noted that these theoretical expectations for the effects of affective polarization on health identify mechanisms involving attitudes of individuals. Namely, in a more affectively polarized environment, a greater number of individual partisans will experience negative emotions in response to the out-party, and such emotions will be more extreme, which may lead to greater stress. At the same time, it is plausible that the polarization of the sociopolitical environment can influence health independently from and simultaneously with its manifestation in individual-level partisan negative emotions. For instance, a broader atmosphere of toxicity in media and political discourse could cause stress regardless of one’s personal orientation toward the political parties. Such effects are likely to be indirect, operate through more complicated mechanisms, and require the operationalization of more abstract concepts compared to the potential influence of individual-level emotive responses on stress and health. Given these considerations, this article proceeds by focusing on the latter. In light of the theoretical links between affective polarization, stress, and health, the first hypothesis tested in this article logically follows:

*Hypothesis 1*: There is a direct negative relationship between affective polarization and health.

#### Hypothesizing a positive relationship: affective polarization as a mobilizing agent

At the same time, affective polarization may positively influence health through political engagement. This indirect effect is predicated on two distinct relationships described in relevant academic literature. First, affectively polarized attitudes have been found to be a powerful motivator of political participation. As Iyengar and Krupenkin (2018) demonstrate, negative sentiment toward one’s political foes is strongly associated with an increased propensity to vote and carry out other election-related activities. In fact, according to the authors, hostility toward political opponents has, in recent years, become a stronger catalyst for engagement than support for one’s own party because individuals may be galvanized by a desire to triumph over those they dislike. There is evidence that this mobilization is driven by partisan emotional responses. Anger toward the out-party or its standard-bearer appears to consistently incite political participation, and anxiety and fear about the consequences of one’s political opponents gaining power may stimulate engagement in less costly or less effortful political activities (Groenendyk and Banks 2014; Valentino et al. 2011; Valentino and Neuner 2017).

Of course, it is plausible that causality operates in the reverse direction such that individuals develop increasingly partisan attitudes after they become politically active. Although there is a dearth of research that compares the relative contribution of each pathway of causality, a number of studies that employ experimental and longitudinal methods provide some indication that political participation follows the negative emotions that can be inspired by the opposing party. For example, Valentino et al. (2011) conducted an experiment in which participants were randomly primed with information meant to elicit different emotional states and were subsequently asked about their intentions to engage in a range of political behaviors. The authors found that those primed to feel anger were more likely to report greater intention to participate in political activities compared to a control group with no exposure to emotional content. Based on a different experiment, Wojcieszak et al. (2016) contend that those who are presented with news articles that affirm their political attitudes are more likely to report intentions to carry out political activities and that this association is partially mediated by feelings of anger. This suggests that when partisans encounter such news, they may experience a similar emotional reaction and be impelled into action. Similarly, Hasell and Weeks’s (2016) analysis of panel data suggests that when partisans consume pro-attitudinal news during an election, they experience anger toward the opposing party’s candidate, which motivates them to post political content online. In sum, existing literature provides a sufficient basis to anticipate that individuals will become more politically active after experiencing emotive responses to the out-party. Thus, the following hypothesis is proposed:

*Hypothesis 2*: Affective polarization is positively associated with political engagement.

Second, if there exists an indirect positive effect of affective polarization on health through political engagement, one would expect participation to be positively associated with health. This hypothesis follows a growing literature that links civic engagement—or one’s involvement in their community and the activities they carry out to shape its future (Adler and Goggin 2005)—to a range of beneficial health outcomes and measures of well-being. Three theoretical mechanisms through which civic engagement may improve health have emerged out of this body of work (for an overview, see Wray-Lake et al. 2019). First, it could bolster individuals’ social networks because involvement in collective projects could connect them to others with similar interests. These new or strengthened social ties may protect health by providing greater access to health information through others, exposing one to more pressure to carry out positive health behaviors, and yielding greater social support, which can mitigate stress (Folland 2007; Kawachi and Berkman 2001). Second, civic engagement may produce positive psychological benefits, like an increased feeling of self-efficacy, a stronger sense of empowerment and purpose, and decreased feelings of loneliness (Ballard, Hoyt, and Pachucki 2019). Such sentiments could protect against stress when an individual encounters difficult circumstances given that improved sense of control and self-esteem have been found to serve as stress buffers (Thoits 2010). Third, it could have direct, health-protective physiological effects on individuals who are civically active. One study found that children randomly assigned to take part in weekly volunteering developed lower cholesterol, lower body mass index, and fewer markers of stress compared to the control group (Schreier, Schonert-Reichl, and Chen 2013).

As a subtype of civic engagement, political participation could benefit health for the same reasons, drawing individuals deeper into associational life or facilitating a more optimistic view of one’s capacity to shape their environment. Indeed, some contributors to the existing body of literature about civic engagement and health acknowledge that the proposed mechanisms linking civic engagement more broadly to well-being could apply to political participation even if political behaviors differ from other civic actions, like volunteering (e.g., Ballard et al. 2019; Ballard and Syme 2016; Wray-Lake et al. 2019).

To be sure, some may argue that political participation could undermine health if it exposes individuals to greater stress. This may occur if those who are politically active spend more time thinking about social problems and the slow pace of positive social change (Ballard et al. 2019). Yet current evidence suggests that any negative psychological effects caused by political engagement may be limited to contentious political activities, and even then, results of relevant studies that utilize panel data are mixed. For example, Ballard et al. (2019) found that those who reported attending a rally or march in young adulthood were more likely to engage in risky health behaviors later in life, which could suggest greater stress due to participation in activism. In contrast, Boehnke and Wong’s (2011) analysis of German panel data shows that participants in the West German peace movement opposing nuclear rearmament demonstrated better mental health later in life compared to nonactivists. Wray-Lake et al. (2019), meanwhile, found a relationship between nonelectoral political behaviors and depressive symptoms that, although statistically significant, was very small. Given this literature, there is little reason to adjust the theoretical expectation that political participation, like other forms of civic engagement, is protective of health. The third hypothesis tested in the present study logically follows:

*Hypothesis 3*: Political engagement is positively associated with health.

## Data and Methods

### Data

This study analyzed survey data collected as part of Pew Research Center’s American Trends Panel.^4^ The panel, which started in 2014, has been conducted multiple times per year, with each wave typically focusing on a unique subject. The study was designed so that each wave could be generalized to the wider population of adults living in the United States because each survey includes its own sampling weight based on the subset of individuals from the total pool of panel members that participated. For the main analysis, this article examined data from Wave 16 (n = 4,685), which was fielded between April 5, 2016, and May 2, 2016. This wave was selected because it included measures of health, political participation, and emotional responses to the out-party.

### Measures

#### Affective polarization: partisan emotive response

Affective polarization was measured at the individual level through three binary-response survey items that assessed negative emotive reactions to the out-party. Specifically, respondents reported whether the Democratic Party and Republican Party made them feel frustrated, angry, and afraid. As discussed previously, affective polarization may be multidimensional, in which case, focusing on these indicators alone is a limitation of the study design. Yet of any dimension of the construct, partisan emotive responses were most suitable for testing the hypotheses proposed previously given the theoretical connection between these negative emotions, health, and political participation. For each of the three emotional reactions, a new variable was created to capture those only toward the out-party for participants who identified as Democrats or Republicans and who were independent but reported leaning toward one or the other party. Partisan leaners were included to maintain consistency with existing research because many studies utilize this coding approach (e.g., Druckman and Levendusky 2019; Iyengar and Krupenkin 2018; Mason 2018a), and true leaners (n = 149) were coded as missing.

#### Political participation

Political participation was measured based on participants’ reported engagement in seven activities. These were (1) attending a political rally, speech, or campaign event; (2) working or volunteering for a political party, candidate, or campaign; (3) being an active member of a group—excluding a political party—that seeks to influence government or policy; (4) contacting an elected official; (5) contributing money to a political candidate or campaign; (6) displaying campaign paraphernalia, such as a poster, bumper sticker, clothing, or a button; and (7) posting about one’s support for a campaign on social media. Participation in each of these behaviors was measured through separate questions, and for every activity, respondents indicated as a binary response whether they carried out the behavior in the year prior to the date in which they completed the survey.^5^

#### Health

Self-rated health was used to capture health status, in which participants were asked, “Would you say that in general your health is excellent, very good, good, fair, or poor?” These responses were recoded so that the “poor” response was the lowest value and “excellent” was the highest. This instrument is among the most frequently utilized in health research and is widely viewed as a valid global measure of one’s overall health status (Jylhä 2009). Although it is often included alongside other measures of health as an indicator of its subjective dimension, it holds value when used as a single measure of the construct as well. If one accepts the World Health Organization’s (2020:1) definition of health as “a state of complete physical, mental and social well-being and not merely the absence of disease or infirmity,” the self-rated health survey item is appropriate because it compels respondents to offer such a broad assessment of their overall well-being. Notably, self-rated health is associated with important objective health outcomes, including mortality risk (DeSalvo et al. 2006) and a wide range of physical health conditions (Latham and Peek 2012). In the analysis presented in the following, self-rated health was analyzed as a continuous variable because it was more amenable to correcting for measurement error. However, the results were substantively similar when treating self-rated health as an ordered categorical variable (see Table S1 in the online version of the article).

#### Control variables

A number of covariates were included to account for potential sources of omitted variable bias. First were basic demographics (reference categories appear first in parentheses): sex (female or male), race-ethnicity (white non-Hispanic, black non-Hispanic, Hispanic, or other race-ethnicity), age (18–29, 30–49, 50–64, or 65+), education (less than high school diploma, high school/equivalent, some college/two-year degree, or four-year degree or higher), and income, which was made up of nine categories of income bands, from less than $10,000 per year to $150,000 or more. Continuous variables, age and income, were available only in categorical form in the data set. Aside from these demographics, marital status (not married/partnered or married/partnered) was also included as a covariate in light of existing research showing that it may lead to greater political engagement (Stoker and Jennings 1995) and improved health (Liu and Umberson 2008). In addition, respondents’ health insurance status (not covered by health insurance or covered) was included in the equation predicting health to account for the possible role of differences in access to health care via ownership of insurance.^6^

### Model Specification

Structural equation modeling (SEM) was utilized to construct a model that evaluated the hypotheses derived previously. SEM provided the necessary tools to do so because it allows one to estimate simultaneous equation models that parse the relationships between multiple variables. In this case, it enabled the decomposition of political participation from the affective polarization–health relationship. Furthermore, SEM provides a means for one to introduce latent variables of interest through measurement models that control for measurement error, which, when unaccounted for, can confound associations of interest. This was particularly advantageous here given the focus on more abstract concepts. In addition, incorporating latent variables using measurement models was beneficial because this approach does not assume that each indicator is related to the same degree to the latent variable. Instead, they allow for differing strengths of associations between the observed indicators and the latent variable.

**Figure 1.**
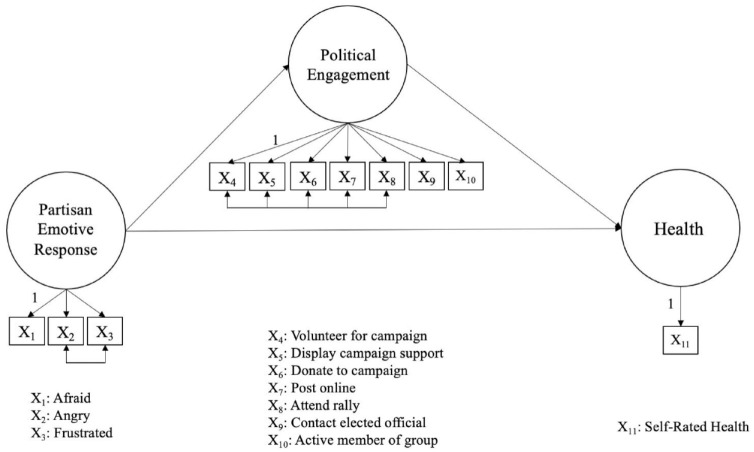
Path Diagram of Structural Equation Model. *Note*: This figure depicts the path diagram of the main model estimated here. Rectangles are observed indicators, and circles are latent variables. Single-headed arrows are direct effects between variables, and straight line segments with short arrows are correlations. Error terms and observed exogenous covariates are not depicted.

In the present study, measurement models were estimated to incorporate latent variables reflecting each of the three constructs of interest: partisan emotive response, political participation, and health. For partisan emotive response and political participation, measurement models were included using each of the indicators described previously. For the former, the fear partisan response was used as the scaling indicator, with the factor loading set to 1. The residual variances of the anger and frustration variables were allowed to correlate because the emotional responses may be similar enough that they could be associated outside of their relationship to the underlying latent variable. For the political engagement variable, campaign volunteering was used as the scaling indicator, and residual variances for the indicators about election-related political engagement were allowed to correlate.^7^ Finally, for health, a single indicator latent variable was estimated with self-rated health. To account for measurement error, the residual variance of the self-rated health indicator was fixed to (1 − ρ_zz_) × σ_z_^2^, in which ρ_zz_ represents the reliability of self-rated health and σ_z_^2^ is its sample variance (Hoyle 2013). The reliability of self-rated health was set with an a priori value of .5, following Bollen et al. (2021).^8^ The direct and indirect effects of affective polarization on health were estimated by regressing the endogenous latent variables on the relevant predictors, as depicted in the path diagram presented in Figure 1. Error terms of the exogenous observed covariates were allowed to correlate.

The model was estimated using Mplus, Version 8.7. The diagonally weighted least squares estimator (WLSMV) with theta parameterization was used to derive the model parameters. This approach was well suited because it does not assume that indicators come from a normal distribution, which was automatically violated by the categorical endogenous variables in the model. With this estimator, coefficients for categorical endogenous variables were estimated using a probit link. The Mplus default for missing data in models with WLSMV and exogenous covariates was used whereby cases with missing data on a covariate were listwise deleted, those with missing data on endogenous variables were pairwise deleted, and those with missing on all variables except observed covariates were dropped. This resulted in the complete exclusion of 177 cases from analysis, 3.8% of the full sample.^9^ Analyses were conducted with the survey weight included in the data set.

## Results

Univariate descriptive statistics of observed indicators are presented in Table 1. Consistent with narratives of a rising tide of affective polarization in the United States, negative partisan emotions were relatively common among survey respondents. Of participants, 43.3%, 56.0%, and 47.9% reported feeling angry with, frustrated with, and afraid of the opposing party, respectively. By contrast, reported political participation varied widely across behaviors, in which participants were least likely to work or volunteer for a campaign or candidate (4.8%) and most likely to post about their support for a candidate online (25.8%). As a general rule, participants were more likely to engage in activities that required less sustained effort. Finally, the sample average self-rated health was 3.318. Results of the measurement models for the three latent variables are presented in Appendix Table A1 and show consistent, positive, statistically significant associations between the latent variables and their indicators, a sign that indicators were well suited to their latent variables.

**Table 1.** Weighted Descriptive Statistics of Observed Variables with Data from the Pew American Trends Panel (2016).

	Mean	SD	Minimum	Maximum	Count
Polarization: angry	.433	.496	0	1	4,517
Polarization: frustrated	.560	.496	0	1	4,517
Polarization: afraid	.479	.500	0	1	4,517
Participation: campaign	.048	.214	0	1	4,641
Participation: display	.145	.352	0	1	4,641
Participation: donate	.146	.353	0	1	4,641
Participation: post online	.258	.438	0	1	4,641
Participation: attend rally	.101	.301	0	1	4,641
Participation: contact official	.183	.386	0	1	4,641
Participation: group member	.087	.282	0	1	4,641
Self-rated health	3.318	1.003	1	5	4,660
Male	.482	.500	0	1	4,685
Republican	.459	.498	0	1	4,536
Income	4.646	2.479	1	9	4,602
Education: less than high school	.109	.312	0	1	4,685
Education: high school	.262	.440	0	1	4,685
Education: some college/2-year degree	.326	.469	0	1	4,685
Education: college or more	.302	.459	0	1	4,685
Married or partnered	.558	.497	0	1	4,651
Age: 18–29	.209	.407	0	1	4,681
Age: 30–49	.331	.471	0	1	4,681
Age: 50–64	.265	.442	0	1	4,681
Age: 65 or older	.194	.396	0	1	4,681
Race-ethnicity: white non-Hispanic	.661	.473	0	1	4,636
Race-ethnicity: black non-Hispanic	.115	.319	0	1	4,636
Race-ethnicity: Hispanic	.145	.352	0	1	4,636
Race-ethnicity: other race-ethnicity	.079	.270	0	1	4,636
Covered by health insurance	.877	.329	0	1	4,644

The full results of the structural model, which relates the direct effects between the latent variables and observed covariates, are presented in Table 2. This table shows the associations between each predictor and the two primary endogenous variables of interest: health and political engagement. Given that both are continuous latent variables, the unstandardized coefficients are interpreted as linear regressions. For continuous predictor variables, standardized coefficients show the standard deviation change in the outcome for each standard deviation increase in the predictor. For the dichotomous covariates, standardized coefficients show the standard deviation difference in the outcome between the reference group and the category coded as 1.

**Table 2.** Structural Equation Model Regression Results with Data from the Pew American Trends Panel (2016).

	Political Engagement		Health	
	Estimate	Standardized	Estimate	Standardized
Partisan emotive response^a^	.552*** (.139)	.363*** (.050)	−.196** (.070)	−.206** (.065)
Political engagement^a^	–	–	.090* (.035)	.144** (.055)
Male	.142* (.068)	.127* (.057)	.073 (.046)	.104 (.066)
Income^a^	.024 (.016)	.053 (.036)	.073*** (.011)	.257*** (.039)
High school	.510** (.185)	.455** (.150)	.097 (.099)	.138 (.141)
Some college	.618*** (.169)	.552*** (.134)	.180 (.098)	.257 (.139)
College	1.002*** (.202)	.894*** (.137)	.321** (.104)	.458** (.148)
Married/partnered	.084 (.077)	.075 (.067)	−.049 (.052)	−.071 (.074)
Age 30–49	−.182 (.110)	−.163 (.094)	−.193** (.072)	−.275** (.102)
Age 50–64	−.050 (.104)	−.045 (.092)	−.396*** (.070)	−.564*** (.098)
Age 65+	.142 (.110)	.127 (.098)	−.314*** (.074)	−.448*** (.104)
Black	.026 (.131)	.023 (.117)	.034 (.076)	.048 (.109)
Hispanic	−.118 (.130)	−.105 (.117)	−.078 (.076)	−.111 (.108)
Other race	−.064 (.116)	−.057 (.103)	−.091 (.093)	−.130 (.132)
Insured	—	—	−.106 (.078)	−.151 (.112)
*R* ^2^	.232		.223	

*Note*: All categorical covariates standardized on *y* only. Standard errors are in parentheses. *n* = 4,508. Model fit indices: χ^2^ = 350.739, *df* = 149, *p* < .0001. Root mean square error of approximation = .017; comparative fit index = .929; Tucker-Lewis index = .906; standardized root mean squared residual = .059; Bayesian information criterion = −902.889.

a Continuous variables standardized on *x* and *y*.

* *p* < .05, **p < .01, ***p < .001 (two-tailed test).

These results yield support for each of the three hypotheses proposed previously. Regarding the direct effect between partisan emotive response and health, there was a statistically significant, negative association. For each standard deviation increase in partisan negative emotions, health decreased by .206 standard deviations (*p* = .001) net of other factors. As to the other two hypotheses, for each 1 SD increase in partisan emotive response, there was a .363 SD increase in political participation (*p* < .001). In turn, health increased by .144 SD for each 1 SD increase in participation (*p* = .009). Table 3 reports the size and significance of the indirect effect; for each 1 SD increase in partisan negative emotions, health increased by .052 SD through political participation (*p* = .027). This suggests that political participation suppresses the relationship between partisan emotive response and health; if not for increased engagement, the negative association between partisan emotions and health would be greater. Even so, the relatively larger direct effect indicates that these emotional responses are, as a whole, correlated with worse health.^10^

**Table 3.** Decomposition of Effects with Data from the Pew American Trends Panel (2016).

	Unstandardized			Standardized		
	Direct Effect	Indirect Effect	Total Effect	Direct Effect	Indirect Effect	Total Effect
Partisan emotive response → health	−.196** (.070)	.050* (.024)	−.146* (.058)	−.206** (.065)	.052* (.024)	−154** (.054)

*Note*: Standard errors are in parentheses. Indirect effect was calculated using the delta method. *n* = 4,508.

* *p* < .05, ** *p* < .01 (two-tailed test).

### Auxiliary Analysis and Robustness Checks

A number of auxiliary analyses were conducted to better understand the nature of the relationships described previously (see the Appendix in the online version of the article). First, the main model was estimated using a self-rated health measure gathered in Wave 28 of the panel, which was fielded in August 2017, more than a year after the collection of the survey with the polarization and engagement items. This analysis was useful given its temporal precedence; with a health measure gathered later than the predictors, the risk of simultaneity bias was mitigated. Because approximately 20% of the sample that participated in Wave 16 did not take part or rate their health in Wave 28, Mplus was used to multiply impute (with 150 imputations) the missing data for only the later health measure to maintain statistical power. Thus, this analysis utilized data from the same survey respondents who rated their health in Wave 16. The results of this model are presented in Table S2 in the online version of the article; health in Wave 28 decreased by .154 SD for each standard deviation increase in partisan emotive response (*p* = .022) and increased by .130 SD for each standard deviation increase in political engagement (*p* = .020).

Some might argue that the results of the main analysis were confounded by the strength of one’s party identity. For instance, it may be that one’s partisan strength is associated with their partisan emotive response, and if it is also correlated with the endogenous variables, the results could be skewed. To account for this possibility, the main model was reestimated with an additional exogenous dichotomous variable reflecting whether the respondent indicated a strong affiliation with their party (see Table S3 in the online version of the article). The coefficients of partisan emotive response and participation in the health equation were attenuated in this model but were still significant.

Finally, the main model was variously reestimated to assess the robustness of the results to alternative methodological decisions. First, the model was estimated assuming a lower and higher reliability of self-rated health in which the reliability was set to .4 and .6, respectively (see Table S4 and Table S5 in the online version of the article). Second, due to the relatively low *R*^2^ value of the frustration indicator in the partisan emotive response latent variable, the model was respecified without the indicator and without correlated residuals between indicators (see Table S6 in the online version of the article). Across these specifications, the results did not drastically change the latent variable regression coefficients.

## Discussion

Political polarization in America today is marked by animosity and distrust. Affiliation with or sympathy toward one major party brings with it a collection of generalizing hostile attitudes toward those on the opposite side of the partisan divide that extend far beyond policy disagreements. Indeed, party identity appears to serve as a critical boundary along which sentiment and perceptions of worth are organized such that polarization is a vector for hostility and, increasingly, violence. There is little reason to believe that affective polarization will be mitigated in the near future so long as the media environment remains fractured, and many political representatives have little incentive to soften their rhetoric when the main threat to their reelection is a primary challenge from their own party.

This article has argued that these trends may exert an impact on health that, although heterogeneous, is net negative. On one hand, the analysis presented here shows a direct negative association between partisan emotive responses and health. This relationship was hypothesized to operate through stress, in which affective polarization was theorized to act as a sociopolitical stressor, namely, by eliciting injurious emotions in reaction to the opposing party. On the other hand, negative partisan emotions were found to be positively associated with health through political engagement. This indirect effect is consistent with existing theory in which it is expected that affective polarization will generate greater motivation to engage politically and that such engagement will proffer health-protective benefits. As demonstrated, the direct negative relationship between partisan emotive response and health outweighs the indirect effect, suggesting that, cumulatively, affective polarization undermines health despite its capacity to catalyze political engagement.

These results imply that it may be necessary to revisit and qualify preexisting notions about the benefits of civic engagement. The norm has generally been to view an engaged population as an unequivocally positive outcome. In particular, following de Tocqueville’s ([1835, 1840] 1990) influential account of the role of associational life in early American democracy, an active public has been assumed to be a credit to democratic systems. Yet some researchers have argued that this statement must be conditioned because not all forms of participation in all contexts necessarily produce social benefits (e.g., Paxton 2002, 2007). This article extends the latter line of reasoning to health. As mentioned, some have argued that individuals accrue health benefits through their engagement in public affairs. However, these effects may be limited if such activity occurs within a polarized environment characterized by a politics laden with anger, fear, and frustration. In other words, political participation may exact hidden costs in factional contexts, like the contemporary United States.

At the same time, the analysis indicates the possibility that political participation serves as a stress buffer that mitigates the negative relationship between partisan disposition and health. Indeed, if not for the increased political participation that comes along with an affectively polarized society, the total negative relationship between affective polarization and health would be larger. According to Wheaton (1985), the existence of a suppression effect, in which the inclusion of an intervening variable (political engagement) strengthens the primary negative relationship of interest (that between affective polarization and health), can be interpreted as evidence of stress buffering. In this vein, the improved sense of self-efficacy or increased social capital theorized to be associated with political participation could serve as coping resources that equip individuals with tools to withstand the stress that accompanies partisanship.

There were a number of limitations of the research design that threaten these conclusions. First, the main analysis was based on a cross-sectional data set, which did not allow for strong causal claims to be made. Although the data were collected as part of a panel, the measures of affective polarization and political engagement utilized here were not repeated and gathered concurrently with later measures of health. Although the results were corroborated with a model that included a later measure of health to mitigate the risk of simultaneity, this approach did not eliminate omitted variable bias, if it was a factor that influenced results, nor did it account for possible reverse causality between partisan emotive responses and political engagement. Second, despite the study’s strength in addressing measurement error in self-rated health, the indicator is an ambiguous one that offers little indication of which particular health consequences follow from affective polarization given that it is correlated with such a wide range of health outcomes (Latham and Peek 2012). In addition, it is plausible that there was measurement variance for self-rated health by political ideology. Whether such variance exists and its potential implications for the findings of this study must be adjudicated by future research. Finally, this study only measured the emotive dimension of affective polarization, not the others described previously. If the true factor structure of affective polarization is multidimensional, the present study may be limited in its measurement of only a single aspect of the construct.

Future work about the health effects of affective polarization can build on the present study by pursuing a number of alternative research designs. First, subsequent research will benefit from including more specific and a wider variety of health measures. Doing so will yield greater insight into the mechanisms through which affective polarization and health are related and the specific impacts it may exert on health. There may be particular value in examining nonsubjective health measures that could differentiate physical from psychosocial health effects given the possibility that affectively polarized attitudes themselves could indicate poor psychosocial health. Second, a proper longitudinal study with consistent, concurrent measurement of polarization, political engagement, and health will contribute significantly to evaluating the internal validity of the claims offered in this article. This is because analyses of such data will enable researchers to better account for unobserved heterogeneity and test the directionality of associations. Third, future studies should determine whether this relationship varies across party affiliation and over time. Such research could clarify whether the findings discussed here are unique to the 2016 election, which may have been particularly charged and stressful, or to particular parts of the election cycle as partisan tensions become inflamed or subside. Finally, this study focused on the manifestation of affective polarization in individual attitudes and its effects on health. Future projects should consider whether a polarized social environment has an independent, indirect influence on health aside from generating individual-level partisan attitudes.

## Conclusion

Partisan antipathy is perhaps among the most vexing problems facing contemporary American democracy, not merely because it threatens the functioning of the political system but also due to its nonpolitical consequences. As this article has proposed, Americans’ diminished health and well-being may be one of them, under the logic that such a hostile political milieu serves as a persistent source of stress. Thus, this article provides further evidence that the sociological truism that the social environment shapes population health extends to the politico-cultural context as well.

## Supplemental Material

sj-docx-1-hsb-10.1177_00221465221075311 – Supplemental material for Resentment Is Like Drinking Poison? The Heterogeneous Health Effects of Affective Polarization Click here for additional data file. Supplemental material, sj-docx-1-hsb-10.1177_00221465221075311 for Resentment Is Like Drinking Poison? The Heterogeneous Health Effects of Affective Polarization by Micah H. Nelson in Journal of Health and Social Behavior

## Appendix: Measurement Model

**Table A1.** Results of Measurement Model with Data from the Pew American Trends Panel (2016).

	Latent Variable	Factor Loading (Standardized)	*R* ^2^
Afraid	Affective polarization	.594*** (.059)	.353
Frustrated	Affective polarization	.289*** (.059)	.084
Angry	Affective polarization	.575*** (.058)	.330
Campaign	Political participation	.746*** (.042)	.557
Display	Political participation	.588*** (.041)	.345
Donate	Political participation	.660*** (.038)	.435
Post online	Political participation	.597*** (.039)	.356
Attend rally	Political participation	.653*** (.038)	.427
Contact official	Political participation	.740*** (.033)	.547
Group member	Political participation	.730*** (.032)	.532
Self-rated health	Health	.707*** (.013)	.500

*Note*: Standard errors are in parentheses. The factor loadings show the relationship between each indicator and its respective latent variable. *n* = 4,508.

*** *p* < .001 (two-tailed test).

## Notes

1. Although, see Smith, Hibbing, and Hibbing (2019), who examine whether Americans perceive that “politics” undermine their health. See also Nayak et al. (2021), who assess whether the perception that partisanship changed after the 2016 election was associated with health outcomes. Finally, see Panagopoulos et al. (2021), who use measures that assess how politically isolated individuals are in their communities.

2. There is disagreement as to whether the American public has become more ideologically polarized over time. This does not invalidate the claim that affective polarization has increased, given that ideological and affective polarization are distinct, and some argue that partisan animus has intensified even as party members have not diverged in their policy preferences (Lelkes 2018).

3. There is some debate about the strength of this finding; Klar, Krupnikov, and Ryan (2018) argue that social distance questions about marriage may be afflicted by measurement error.

4. The opinions expressed herein, including any implications for policy, are those of the author and not of Pew Research Center.

5. For both the partisan emotive response and political participation items on the survey, respondents were shown one tick box for each item and asked to “Check all that apply”; therefore, for individual items, both missing and negative responses appeared the same in the data set. However, because each module included a box for “None of these,” those who skipped the entire module could be differentiated from those who offered legitimate negative responses for all items. To filter out skipped items, participants who did not check any box in the module, including “None of these,” were coded as missing: 19 for the polarization variables and 44 for the political participation variables.

6. Political affiliation (Democrat or Republican) was excluded for the sake of parsimony; its inclusion worsened model fit and did not drastically change the primary coefficients of interest (see Table S7 in the online version of the article).

7. It is debatable whether reported engagement in political behaviors should be treated as formative indicators of the latent variable (in which, change in the indicators leads to change in the latent variable) rather than effect indicators, as is the case here. Although the use of formative indicators is not feasible here because the resulting model would not be identified, a case in favor of effect indicators can be made, following Bollen and Bauldry (2011). First, the political engagement variables were all positively correlated. Second, omitting any single engagement indicator from the model did not drastically change the meaning of the latent variable.

8. In their study, the authors find that the reliability of self-rated health generally varies between .5 and .6. The lower end of this range was used here in the main analysis for a more conservative tack toward the risk of measurement error.

9. The large number of categorical indicators in the model prevented the use of the full information maximum likelihood (FIML) approach for handling missing data. However, the model was additionally estimated using multiple imputation (with 150 imputations), which requires the same assumptions about missingness as FIML and produces similar estimates. The results of this model were similar to those of the main analysis with respect to the three hypotheses.

10. Fit statistics for the main model are presented in Table 2 and indicate adequate model fit. Both the root mean square error of approximation and the standardized root mean squared residual fall under their good-fit cutoffs, .05 and .08, respectively (Hu and Bentler 1999). Furthermore, the Bayesian information criterion indicated very strong model fit (Raftery 1995), in which a more negative result indicates superior fit. In judging the validity of the model, there is reason to weight the last measure most strongly in light of evidence of its superior performance in identifying the best-fitting model in large samples relative to the aforementioned indices (Bollen et al. 2014). Although the chi-square test indicates a mismatch between the observed and model-implied means and covariances, the test is extremely sensitive to minor deviations from perfect in large samples such as here. Finally, the Tucker-Lewis index and comparative fit index are just under Hu and Bentler’s (1999) suggested .95 good-fit cutoff. Yet, given the large number of model parameters, these fit statistics are acceptable (Weston and Gore 2006).
