# Supplementary material for: Resentment Is Like Drinking Poison? The Heterogeneous Health Effects of Affective Polarization
Source: J Health Soc Behav. 2022 Feb 11;63(4):508–24. doi: 10.1177/00221465221075311 (PMC9716484; doi:10.1177/00221465221075311)
Supplement: sj-docx-1-hsb-10.1177_00221465221075311 – Supplemental material for Resentment Is Like Drinking Poison? The Heterogeneous Health Effects of Affective Polarization [file sj-docx-1-hsb-10.1177_00221465221075311.docx]

**Journal** of **Health**

and **Social Behavior**

OFFICIAL JOURNAL OF THE AMERICAN SOCIOLOGICAL ASSOCIATION

**ONLINE SUPPLEMENT**

**to article in**

Journal of Health and Social Behavior

**Resentment is Like Drinking Poison? The Heterogeneous Health Effects of Affective Polarization**

**Micah H. Nelson**

*University of North Carolina at Chapel Hill*

APPENDIX S. Robustness Checks and Auxiliary Analysis

| Table S1 Main Model Using Ordinal Self-Rated Health Variable (Probit) with Data from the Pew American Trends Panel (2016) | | | | |
| --- | --- | --- | --- | --- |
|  | **Political Engagement** | | **Self-Rated Health** | |
|  | Estimate | Standardized | Estimate | Standardized |
| Partisan emotive response† | .547***  (.137) | .363***  (.050) | –.222**  (.080) | –.154**  (.048) |
| Political engagement† | – | – | .103*  (.040) | .107**  (.041) |
| R-square | .233 | | .121 | |
| Model fit indices  𝜒^2^ =350.834, df = 149  RMSEA = .017; CFI = .929; TLI = .906; SRMR = .058; BIC = –902.794 | | | | |

*Note:* Standard errors are in parentheses; †Continuous variables standardized on x and y; *n* = 4,508

**p* < .05; ** *p* < .01; *** *p* < .001 (two-tailed)

| Table S2 Partial Results: Predicting Later Health Measure with Data from the Pew American Trends Panel (2016) | | | | |
| --- | --- | --- | --- | --- |
|  | **Political Engagement** | | **Health (Wave 28)** | |
|  | Estimate | Standardized | Estimate | Standardized |
| Partisan  emotive response† | .531***  (.138) | .364***  (.050) | –.153*  (.073) | –.154*  (.067) |
| Political engagement† | – | – | .088*  (.039) | .130*  (.056) |
| R-square | .232 | | .229 | |
| Model fit indices  𝜒^2^ =336.069, df = 149  RMSEA = .017; CFI = .935; TLI = .913; SRMR = .058; BIC = –917.625 | | | | |

*Note:* Results are averaged over 150 datasets. Standard errors are in parentheses; †Continuous variables standardized on x and y; *n* = 4,510

**p* < .05; ** *p* < .01; *** *p* < .001 (two-tailed)

| Table S3 Partial Results: Including Partisan Strength with Data from the Pew American Trends Panel (2016) | | | | |
| --- | --- | --- | --- | --- |
|  | **Political Engagement** | | **Health** | |
|  | Estimate | Standardized | Estimate | Standardized |
| Partisan  emotive response† | .587***  (.159) | .340***  (.052) | –.191**  (.073) | –.195**  (.065) |
| Political engagement† | – | – | .071*  (.031) | .124*  (.054) |
| Strong partisan | .383***  (.091) | .313***  (.058) | .059 (.047) | .085  (.068) |
| R-square | .231 | | .222 | |
| Model fit indices  𝜒^2^ = 426.209, df = 158, p < .0001  RMSEA = .020; CFI = .907; TLI = .877; SRMR = .062; BIC = –901.095 | | | | |

*Note:* Standard errors are in parentheses; †Continuous variables standardized on x and y; *n* = 4,450

**p* < .05; ** *p* < .01; *** *p* < .001 (two-tailed)

| Table S4 Partial Results: .4 Reliability of Self-Rated Health with Data from the Pew American Trends Panel (2016) | | | | |
| --- | --- | --- | --- | --- |
|  | **Political Engagement** | | **Health** | |
|  | Estimate | Standardized | Estimate | Standardized |
| Partisan emotive response† | .549***  (.138) | .363***  (.050) | –.194**  (.070) | –.229**  (.072) |
| Political engagement† | – | – | .090*  (.035) | .160**  (.062) |
| R-square | .232 | | .278 | |
| Model fit indices  𝜒^2^ = 350.737, df =149, p < .0001  RMSEA = .017; CFI = .929; TLI = .906; SRMR = .059; BIC = –902.891 | | | | |

*Note:* Standard errors in parentheses; †Continuous variables standardized on x and y; *n* = 4,508

**p* < .05; ** *p* < .01; *** *p* < .001 (two-tailed)

| Table S5 Partial Results: .6 Reliability of Self-Rated Health with Data from the Pew American Trends Panel (2016) | | | | |
| --- | --- | --- | --- | --- |
|  | **Political Engagement** | | **Health** | |
|  | Estimate | Standardized | Estimate | Standardized |
| Partisan emotive response† | .550***  (.138) | .363***  (.050) | –.194**  (.070) | –.187**  (.059) |
| Political engagement† | – | – | .090*  (.035) | .131**  (.050) |
| R-square | .232 | | .186 | |
| Model fit indices  𝜒^2^ = 350.737, df =149, p < .0001  RMSEA = .017; CFI = .929; TLI = .906; SRMR = .059; BIC = –902.891 | | | | |

*Note:* Standard errors in parentheses; †Continuous variables standardized on x and y; *n* = 4,508

**p* < .05; ** *p* < .01; *** *p* < .001 (two-tailed)

| Table S6 Partial Results: Excluding Frustration Indicator from Affective Polarization with Data from the Pew American Trends Panel (2016) | | | | |
| --- | --- | --- | --- | --- |
|  | **Political Engagement** | | **Health** | |
|  | Estimate | Standardized | Estimate | Standardized |
| Partisan emotive response† | .460***  (.124) | .342***  (.050) | –.153*  (.061) | –.180**  (.062) |
| Political engagement† | – | – | .083*  (.034) | .131*  (.054) |
| R-square | .219 | | .214 | |
| Model fit indices  𝜒^2^ = 319.166, df = 149, p < .0001  RMSEA = .018; CFI = .931; TLI = .905; SRMR = .057; BIC = –934.462 | | | | |

*Note:* Standard errors are in parentheses; †Continuous variables standardized on x and y; *n* = 4,508

**p* < .05; ** *p* < .01; *** *p* < .001 (two-tailed)

| Table S7 Partial Results: Including Party ID with Data from the Pew American Trends Panel (2016) | | | | |
| --- | --- | --- | --- | --- |
|  | **Political Engagement** | | **Health** | |
|  | Estimate | Standardized | Estimate | Standardized |
| Partisan  emotive response† | .547***  (.139) | .364***  (.051) | –.185**  (.070) | –.194**  (.065) |
| Political engagement† | – | – | .086*  (.036) | .135*  (.055) |
| Republican | –.095  (.070) | –.086  (.064) | .127* (.049) | .182*  (.070) |
| R-square | .225 | | .224 | |
| Model fit indices  𝜒^2^ = 384.524, df = 158, p < .0001  RMSEA = .018; CFI = .919; TLI = .893; SRMR = .058; BIC = –940.095 | | | | |

*Note:* Standard errors are in parentheses; †Continuous variables standardized on x and y; *n* = 4,375

**p* < .05; ** *p* < .01; *** *p* < .001 (two-tailed)
